# Impact of Self-Concept, Self-Imagination, and Self-Efficacy on English Language Learning Outcomes Among Blended Learning Students During COVID-19

**DOI:** 10.3389/fpsyg.2022.784444

**Published:** 2022-03-04

**Authors:** Ruihua Chen, Javed Iqbal, Yanghe Liu, Mengmei Zhu, Yi Xie

**Affiliations:** ^1^School of Education, Guangzhou University, Guangzhou, China; ^2^Moray House School of Education and Sport, University of Edinburgh, Edinburgh, United Kingdom; ^3^Guangzhou Academy of Fine Arts, Guangzhou, China

**Keywords:** self-concept, self-imagination, self-efficacy, English language learning outcomes, structural equation modeling

## Abstract

The purpose of the present study was to explore the direct influence of self-concept and self-imagination on English language learning outcomes (ELLO). Furthermore, this study examined the mediating role of self-efficacy in the relationship between self-concept, self-imagination, and ELLO. A survey questionnaire of 21 items was used in this study. We distributed the questionnaire through QR code and collected the data from 2,517 participants who enrolled in blended learning courses at the undergraduate level in Chinese universities. The relationship among the variables was measured through SmartPLS-SEM 3.3.3 (partial least squares structural equation modeling). The outcomes of the present study indicated a direct, positive, and significant connection of self-concept, self-imagination, and self-efficacy with ELLO. Looking at indirect influences, self-concept and self-imagination, positive and significant, influence ELLO through self-efficacy. Thus, self-efficacy was indicated to play a mediating role between self-concept, self-imagination and ELLO. We can conclude that self-concept, self-imagination, and self-efficacy are the main predictors of ELLO in blended learning courses during the pandemic. Additionally, self-concept and self-imagination along with the intervening role of self-efficacy, play a more effective role in improving ELLO. Moreover, this study provided some useful, practical implications, and future research directions.

## Introduction

English language learning outcomes (ELLO) are very critical components of academic progress (Bai and Wang, 2020). In most countries wherein English is a non-native language, it has become a key area for learning along with their native language (Tsui and Tollefson, 2017). Furthermore, English learning through blended learning has become vital during the COVID-19 pandemic (Adedoyin and Soykan, 2020). It works as a tool for the teaching–learning process to maintain social distance standards of procedures to curb this pandemic and limit its spread (Adedoyin and Soykan, 2020; Alrefaie et al., 2020). For this purpose, higher education institutions shifted their teaching practices to a blended learning model (Khan et al., 2021). It was important to know how self-concept, self-imagination, and self-efficacy influence ELLO among students through this model in the COVID-19 pandemic. This situation provoked the authors to investigate the impact of self-concept, self-imagination, and self-efficacy on ELLO among students.

The self-concept is a cognitive process that influences the students’ ELLO (Grilli and McFarland, 2011). In a broader sense, this self-concept provides a significant base to understand self, which refers toward students’ better performance in their study (Chen et al., 2021). Self-concept shows a perception of self in a specific domain of work area such as ELLO (Bong and Skaalvik, 2003; Guo et al., 2021), although, it works for the internal or external frame of reference, which influences students’ personal belief about strong areas wherein they can perform successfully (Grilli and McFarland, 2011). Based on the discussion, it was assumed that self-concept might influence ELLO (Fryer, 2015; Biyikl, 2021; Guo et al., 2021).

The self-imagination concept deals with a student’s perspective for performing a work activity in academia (Grilli and McFarland, 2011). It gives a belief or wishes to the individual to perform a task somewhere else in the future (Murray, 2011). Similarly, self-imagination provides the belief to the students that they can communicate in English with their international friends or colleagues lies in their imagination (Dörnyei, 2009). Similarly, It has been discussed that teachers need to design learning environments and encourage them to engage their self-imagination in learning (Murray, 2011). It is essential to understand how self-imagination improves ELLO through blended learning (Rivers et al., 2021).

Most studies suggested that self-efficacy’s role in learning is needed to explore further (Shin, 2018). Moreover, self-efficacy provides courage to the students that they should be skilful enough to improve ELLO (Erten and Burden, 2014; Chen et al., 2015; Kim et al., 2015; Noorollahi, 2021). It has been discussed that self-efficacy is a domain-specific construct. It works in language learning or the common learning domain. Similarly, students believe that they are strong enough to perform any activity in a specific area and compare themselves where they are weak to perform other tasks (Marsh et al., 2006; Lauermann and ten Hagen, 2021). Therefore, the significant role of self-efficacy in enhancing ELLO is explored in this study.

To the best knowledge of the authors, the literature supports the perspective that the positive relationships between self-concept, self-imagination, self-efficacy, and ELLO contexts have not been sufficiently explored, especially in the COVID-19 pandemic. In line with these views there was a requirement to complementary investigate the implications of self-concept, self-imagination, and self-efficacy on ELLO, especially in blended learning. Therefore, the current study argued how self-concept and self-imagination influence ELLO by using self-efficacy as a mediator variable.

In this paper, we adopted the perspective which was presented in the social learning theory (Zimmerman, 1983). Social learning theory provides the views of how cognitive process interacts to influence individual learning, especially Bandura’s self-efficacy theory (SET), which is a subset of social learning theory in the sense of the cognitive process that plays a role in dealing with learning challenges (Genc et al., 2016). However, the extensive literature review has so far offered simply partial models of relationships that although are statistically acceptable and contribute to understanding the scope of self-efficacy, do not provide a comprehensive vision to the phenomenon proposed in the current study (Erten and Burden, 2014; Chen et al., 2015; Kim et al., 2015). Further, the mediating role of self-efficacy in the relationship between self-concept, self-imagination, and ELLO has not been adequately explored.

For this research gap, the core objective of this study was to shed light on the influence of self-concept and self-imagination on ELLO through blended learning during the COVID-19 pandemic. It is further explained in the next section that this study explores the intervening role of self-efficacy. To understand a broad view of the phenomenon, it also ponders on a number of variables emphasized in the literature as affecting the association between self-concept, self-imagination, and ELLO, namely self-efficacy. In this paper, a quantitative study was conducted in China, comprising 2,517 students enrolled in blended learning courses and applied structural equation modeling to measure a set of research hypotheses.

This paper provides various contributions. First, it enhances the existing literature about the influence of self-concept and self-imagination on ELLO. Second, this study proposed a synthesized research model that integrates a supplementary perspective of the indirect influence of self-concept and self-imagination on ELLO. Third, this study further offers empirical evidence and robust statistical analysis that explains both direct and indirect relationships and helps to understand how self-concept and self-imagination, along with self-efficacy, positively influence the ELLO during the COVID-19 pandemic. Lastly, this study offers insight on how to enhance the potential influence of self-concept and self-imagination application as it demonstrates that self-efficacy positively mediated ELLO. As a whole, this study provides meaningful insights for practitioners and academicians. The above discussion motivates the researchers to address these gaps within the developed research framework and formulate the following research questions.

**RQ 1:** How do self-concept, self-imagination, and self-efficacy influence English language learning outcomes?

**RQ 2:** How does self-efficacy intervene in the relationship between self-concept, self-imagination, and English language learning outcomes?

This study is divided into 7 sections. Section 1 deals with the introduction of this study. Section 2 deals with the research framework and literature review along with hypothesis formulation. Section 3 presents the details of the research methodology. Section 4 provides the details of data analysis and interpretation. Section 5 provides the details of the results’ discussion. Section 6 deals with conclusions, and section 7 presents the implications, limitations, and future research directions.

## Literature Review

### Research Framework

The sudden emergence of COVID-19 pandemic disrupted the higher education system across the world where online learning has become vital, and universities are well aware of its significance during this pandemic (Khan et al., 2021). Various scholars have discussed the practical and theoretical issues related to ELLO. Baloran (2020) addressed the students’ psychological issues such as anxiety, attitudes, and knowledge and coping strategies in blended learning during the COVID-19 pandemic. Similarly, Yang et al. (2021) discussed the role of classroom engagement and activities in English learning as a foreign language outcomes at a higher education level. Some authors discussed pedagogical transformation and technology-mediating role in the relationship between various constructs such as language learning environment, collaborative learning skills, self-directed, and English self-efficacy through blended learning mode during the COVID-19 pandemic (Lian et al., 2021). However, few studies investigated the role of self-concept, self-imagination, and self-efficacy for ELLO through blended learning mode. Therefore, this study investigated the relationships of these constructs through a synthesized research framework.

**FIGURE 1:**
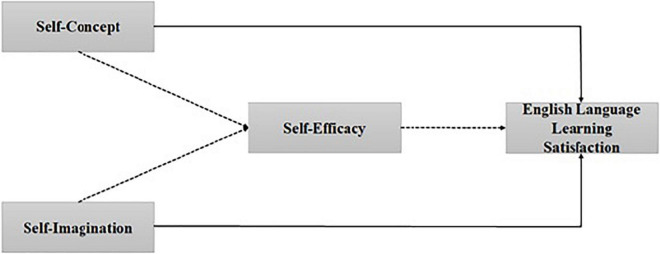
Research model.

The research framework highlights the relationship between self-concept, self-imagination, self-efficacy, and ELLO. Self-concept and self-efficacy are considered as an extended topic among educational psychology scholars. Many studies in this field of educational psychology tried to explore the role of self-concept and self-efficacy in improving ELLO (Henderson et al., 2012; Zhao et al., 2021). Several studies showed that self-concept and self-efficacy have a vital role in improving ELLO (Chao et al., 2019; Bai and Wang, 2020). A self-imagination role for enhancing ELLO is yet to be explored. In this study, self-imagination was studied as a supplementary construct along with self-concept and self-efficacy to improve ELLO. Moreover, various scholars have claimed that socio-cultural theory contends that self-concept and self-efficacy positively correlate with ELLO (Chao, 2013; Ozfidan et al., 2014). Thus, the present study contends that self-efficacy mediates the relationship between self-concept, self-imagination, and ELLO and seeks to explore the mediated effect of self-efficacy in the relationship between self-concept, self-imagination, and ELLO. The present study analyzes these connections empirically and highlights the impact of self-concept and self-imagination on ELLO through self-efficacy in an emerging country, China. This study also supplements previous work by adding the role of self-imagination in improving ELLO. Existing literature explains that self-concept, self-imagination, and self-efficacy have a significant relationship with ELLO. Based on this discussion, the synthesized research model of the study has explained the relationships in the following hypotheses (see Figure 1).

### Self-Concept and English Language Learning Outcomes

Self-concept is one’s perception of behavior and personal attributions (Wylie, 1974; Gecas, 1982; Marsh and Hattie, 1996). In other words, self-concept is referred to as the set of beliefs about oneself (Rogers et al., 1978; Moore and Oosthuizen, 1997). From an educational perspective, some scholars emphasized that self-concept, task-specific or domain-specific (Arens and Jansen, 2016; Oxford, 2017), construct and vary from subject area to subject area such as students’ English language learning self-concept will be potentially different from other learning areas (Waddington, 2019). In the foreign language learning domain, self-concept refers to learners’ self-perception of their positions in the language learning process (Dörnyei and Ushioda, 2009; Mercer, 2011). It has been debated that self-concept is required to focus on student cognitive beliefs which give them ideal courage that they have capabilities to deal in their affective domain (Waddington, 2019).

Many studies have highlighted research frameworks and conceptions to explore the associations between self-concept and ELLO. It has been mentioned that self-concept has a positive and significant relationship with ELLO (Kirmizi, 2015; Chao et al., 2019; Waddington, 2019). The literature consistently argues that self-concept in English domains can predict English language learning (Chao et al., 2019). Abundant studies have provided supporting evidence that self-concept is strongly associated with language learning (Erten and Burden, 2014; Chen et al., 2015; Kim et al., 2015). Research studies discussed that the use of technologies in a language learning environment engaged students more and contributed toward writing self-concept and achievements among students (Chen et al., 2021). Biyikl (2021) argued increased academic self-concept led to improved scores in language-learning strategies such as verbal skills. Rivers et al. (2021), highlighted the role of virtual reality for socially isolated learners during the COVID-19 pandemic, and concluded that online learning solutions is significant for a virtual reality assisted analysis of academic self-concept among online learners at a Japanese university. Similarly, Moyer (2018) explains that self-concept is a key obligation for language learning outcomes. Chen et al. (2015) have provided evidence that academic self-concept is significantly related to learning performance. These arguments suggest that self-concept is positively associated with ELLO, which is predicted in the following hypothesis outcomes:

H1: Self-concept has a positive and significant association with English language learning.

### Self-Imagination and English Language Learning Outcomes

Self-imagination is a cognitive process that derives a person’s imagination and awareness to construct and modify realities. It enhances the memory of the students, even those having a relatively poor memory functioning (Grilli and Glisky, 2011). Most of the studies discussed the importance of self-imagination and its functions, such as self-imagination could elaborate signs based on the knowledge. This knowledge initially stimulates the memories of the past and applies them in the future. It initiates such memorable knowledge and events which occurs in individual mind replications in near-future events (Conway and Loveday, 2015). As Oyserman and Markus (1990) proposed, ideal selves can enhance the regulation of behavior. The function of self-imagination plays an important role in understanding the formation of ideal second language identities and its effects on foreign language learning (Al-Shehri, 2009b). Several scholars investigated the construct of self-imagination in the fields of medical education and psychology to understand its implications on mental and cognitive health. The relationship between self-imagination and mathematics learning was also discussed (Sabirova et al., 2020). The researcher explored the role of imagination in English language learning among students enrolled in the blended self-regulated learning and formal instruction, and the results indicated that self-imagination is positively associated with English language learning (Murray, 2011). Ignatova et al. (2020) explored the language ingredients that were expectedly difficult to learn and asking learners to imagine textual material to enhance language learning. Based on the above discussion, we assumed that self-imagination has a positive and significant role in improving ELLO. Thus, the positive associations between self-imagination and English language learning outcomes are predicted in the following hypothesis:

H2: Self-imagination has a positive and significant influence on English language learning outcomes.

### Self-Efficacy and English Language Learning Outcomes

Self-efficacy is concerned with beliefs in individual capabilities to accomplish given tasks and can influence one’s actions in the executive process. Self-efficacy explains the presumed thoughts and actions (Bandura, 1977, 1997). Most studies explored that learners’ self-efficacy is the main predictor for improving academic performance (Pajares, 2002). It is also discussed that learners with high academic self-efficacy are inclined to seek challenges in learning, while those with low academic self-efficacy usually avoid tasks beyond their self-perceived ability (Chen and Zhang, 2019). Similarly, Wang et al. (2014) proposed that a learner’s self-efficacy in the English language learning domain is one’s perception of expected language learning outcomes according to their former English language learning experience. In recent studies, scholars have increased their interest to explore self-efficacy in the English language learning context (reading, writing, listening, and speaking). For instance, a significant connection between English language learning self-efficacy and writing capabilities have been presented in research on university students in Taiwan (Su et al., 2018). A number of studies explained the relationship between self-efficacy and academic learning (Pajares and Schunk, 2001). Self-efficacy is the most reliable predictor in domain-specific learning. It was also discussed that self-efficacy significantly improves English language performance among students (Anyadubalu, 2010). Another study explored self-efficacy correlating with English language learning (Genc et al., 2016). The prior study successfully introduced English as a foreign language learners’ self-efficacy to theoretical frameworks in the English language learning domain (Su et al., 2018). Existing literature also explains that self-efficacy works a strategy to improve English language performance among students (Anyadubalu, 2010). Lauermann and ten Hagen (2021), highlighted a few other factors that are related to learner’s outcomes such as teachers’ perceived competence and self-efficacy. Noorollahi (2021) explored the connections between self-efficacy and academic learning among Iranian graduate students having a major in English language teaching. These results provided great help to understand the relationship between self-efficacy and ELLO. This study explored the self-efficacy in the domain of English as foreign language learning outcomes in the Chinese context. Thus, the positive associations between self-efficacy and ELLO is assumed in the following hypothesis.

H3: Self-efficacy has a positive influence on the English language learning outcomes.

### Mediating Roles of Self-Efficacy

Though being considered as significant variables in the learning process (Stracke, 2016), limited studies have been done concerning the interrelationships between self-concept, self-imagination, and self-efficacy in Chinese blended learning contexts. For example, some studies have investigated the learning achievement of Chinese ELLO learners who have abundant access to online learning (Su et al., 2018). Information communication technology allows learners to learn the English language in a more innovative way (Bodnar et al., 2016). Technology also increases learners’ flexibility and autonomy in self-regulated ELLO (Lai and Gu, 2011) and enhances their self-efficacy (Henderson et al., 2012; Zhao et al., 2021). Students with awareness of using self-efficacy strategies are more competitive in understanding materials and classroom engagement in blended-learning environments (Chang, 2005). Thus, we assumed the English language learning construct was much needed to explore along with self-concept, self-imagination, and self-efficacy.

The literature explains the positive connections of self-efficacy with English language learning (Genc et al., 2016). However, Bandura (2006) recommended that self-efficacy should be studied in the task-oriented domain in a different context, especially in various educational settings. Some scholars used multidimensions or domain-specific scales to measure language learning self-efficacy (Wang et al., 2013). The role of self-efficacy in the prior study shows that self-efficacy plays an intervening role between emotional states, such as anxiety and English language learning (Woodrow, 2011). Another study attempted to explore language self-efficacy as an individual construct and suggested that it should be exercised as replication in English language self-efficacy at different locations (Su et al., 2018). We found fewer studies that studied the mediating role of self-efficacy in between self-concept, self-imagination, and ELLO. This study measured the mediating role of self-efficacy in the relationship between self-concept, self-imagination, and self-efficacy. Hence, the following hypotheses are put forward:

H4: Self-efficacy has a positive and significant intervening role in the relationship between self-concept and English language learning outcomes.
H5: Self-efficacy has a positive and significant intervening role in the relationship between self-imagination and English language learning outcomes.

### Research Methods

This quantitative study aimed to explore the impact of self-concept, self-imagination, and self-efficacy on ELLO during COVID-19. The relationships between self-concept, self- imagination, and self-efficacy have been generally explored in normal teaching and learning environments, while limited studies are conducted in blended learning contexts. Furthermore, as most universities implemented a blended learning mode during the COVID-19 pandemic, this quantitative study can provide evidence to identify the pedagogical needs of students in blended learning in Chinese contexts. For this purpose, we adopted a questionnaire approach in this study.

### Instrument Development

This study considered self-concept and self-imagination as independent variables, ELLO as the dependent variable, and self-efficacy was used as a mediator variable. A questionnaire was made for this purpose. The questionnaire included two parts. In the first part, respondents’ demographics (gender, year of study, major, latest English test result, and English language learning duration) were collected. Instructions, anonymity, and privacy statements were also presented in this part. In the second part, respondents were required to rate items related to self-concept (5 items), self-imagination (4 items), self-efficacy (6 items), and ELLO (6 items). A 7-point Likert scale was used in the present study. The responses range on all items was from 1 to 7, strongly disagree to strongly agree, respectively. The reliability was measured with the threshold value of 0.70. The convergent and discriminant validity of variables were also measured, explained in Table 1.

**TABLE 1:** Reliability and validity.

Scales	Factor loading	Cronbach’s Alpha	rho_A	Composite reliability	Average variance extracted (AVE)
Self-Concept (SC)		0.878	0.878	0.916	0.733
SC1	0.815				
SC2	0.876				
SC3	0.869				
SC4	0.862				
Self-Imagination (SI)		0.841	0.846	0.887	0.612
SI1	0.689				
SI2	0.792				
SI3	0.781				
SI4	0.841				
SI5	0.800				
Self-Efficacy (SE)		0.964	0.965	0.971	0.849
SE1	0.913				
SE2	0.919				
SE3	0.925				
SE4	0.919				
SE5	0.921				
SE6	0.932				
English Language Learning Outcomes (ELLO)		0.887	0.887	0.915	0.645
ELLO1	0.597				
ELLO2	0.815				
ELLO3	0.854				
ELLO4	0.866				
ELLO5	0.818				
ELLO6	0.837				

### Measures

#### Self-Concept

The items related to self-concept were adapted from the literature (Markus and Wurf, 1987; Schunk and Pajares, 2002; Marsh, 2006). The 7-point Likert scale was used, having a response range from 1 to 7, which indicated their degree of agreement on statements related to self-concept, e.g., “I can apply learned English into my personal and professional life,” “I can apply learned English into my personal and professional life.” The self-concept Cronbach’s Alpha value was (α = 0.878). Therefore, it was concluded that the scale was appropriate.

#### Self-Imagination

The items related to self-imagination was adapted from the work of Taguchi et al. (2009). The 7-point Likert scale was used, having a response range from 1 to 7, which indicated their degree of agreement to statements related to self-imagination. The example items from this section are “l mostly imagine myself living abroad and talking fluently in English” and “speaking English with international friends or colleagues lies in my imagination” The self-imagination Cronbach’s Alpha value was (α = 0.841). Hence, it was concluded that the scale was appropriate.

#### Self-Efficacy

The items related to self-efficacy were adapted from the work of Taguchi et al. (2009) and Shen et al. (2013). The 7-point Likert scale was used, having a response range from 1 to 7, which indicated their degree of agreement to statements related to self-efficacy. The example items from this section are “I can understand class discussions in English,” and “I can understand teacher’s spoken directions for an activity in English.” The self-efficacy Cronbach’s Alpha value was (α = 0.964). Thus, it was concluded that the scale was appropriate.

#### English Language Learning Outcomes

The items related to ELLO were adapted from the work of Dörnyei (2005). The 7-point Likert scale was used, having a response range from 1 to 7, which indicated their degree of agreement to statements related to self-efficacy. The example items from this section are “The atmosphere of online English classes attracts me,” and “English language learning may enhance my communication skills.” The ELLO Cronbach’s Alpha value was (α = 0.887). Thus, it was concluded that the scale was appropriate.

#### Sampling and Data Collection

The stratified random sampling technique was applied to collect the data from universities in different regions (Eastern, Southern, Central, Northern, Northeast, Southeast, Northeast China, as well as Hong Kong, Macao, and Taiwan) in China. The questionnaire was distributed among participants *via* link or QR code. Students enrolled in English language learners’ compulsory courses in the universities of China were the target population of the study. The details of the sample characteristics are discussed in Table 2.

**TABLE 2:** Demographic profile of participants.

Personal attributions	Categories	Frequency (n)	Percentage (%)
Gender	Male	396	15.7
	Female	2121	84.3
	Total	2517	100.0
Major	Social Sciences	1197	47.55
	Art and Humanities	1028	40.84
	Natural Sciences	80	3.17
	Business Sciences	212	8.4
	Total	2517	100.0
Year of study	1^st^	1345	53.4
	2^nd^	953	37.9
	3^rd^	191	7.6
	4^th^	28	1.1
Latest English test result	Under 60	231	9.2
	61-75	772	30.7
	76-85	949	37.7
	Over 86	565	22.5
	Total	2517	100.0
English language learning duration	Less than five years	139	5.5
	Five to ten years	1432	56.9
	More than ten years	946	37.6
	Total	2517	100

#### Participant Demographic Profile

The universities were offering English language courses through blended learning mode during the COVID-19 pandemic. In this situation, we explored the role of self-concept, self-imagination, and self-efficacy’s influence on ELLO among the students enrolled in blended learning courses in the Chinese universities. The majority of the participants were women (84.3% women and 15.7% men); 47.55% of participants belonged to the area of social sciences, 40.84% arts and humanities, 3.17% natural sciences, and 8.4% business sciences; 53.4% were first-year university students, 37.9% in second-year,7.6% in third-year, 1.1% in fourth-year; latest English test result were: 37.7% participants achieved 76%–85%, 30.7% achieved 61%–75%, 22.5% achieved over 86%, and 9.2% under 60%; English language learning duration of participants were as 5.5% less than 5 years, 56.9% 5–10 years, and 37.6% more than 10 years. The collected data were enough to apply PLS_SEM through SmartPLS 3.3.3 statistical software. More details regarding demographic profile of participants is presented in Table 2.

#### Measurement Models

The SmartPLS 3.3.3 statistical software was used for applying the confirmatory factor analysis approaches to measure the measurement models (Shen et al., 2013). According to Sarstedt et al. (2017), variance-based structural equation modeling (VB-SEM) technique is less sensitized as compared to CV-SEM covariance-based structural equation modeling (Sarstedt et al., 2017). The validity and reliability of the scale were examined at the first stage (Bhattacherjee et al., 2008). Table 1 explains the details of the reliability and validity testing of the scales. We measured the reliability index based on factor loading, Cronbach’s alpha, rho_A, and composite reliability indicators. The threshold criteria for these indicators are 0.70, factor loading above 0.50 is also acceptable with an AVE index above 0.50 (Iqbal et al., 2021). The convergent validity was measured by applying the AVE technique. The threshold value for AVE is above 0.50. Table 1 shows that reliability indicators such as factor loading, Cronbach’s alpha, rho_A, and composite reliability indicators are above threshold values; therefore, the scales were reliable to collect the data. The values of AVE on all reflective scales were are above 0.50. Thus, the scale discriminant validity of all scales was appropriate.

The measurement standard for discriminant validity given by Henseler et al. (2014) was criticized by Fornell and Larcker (1981). They said this approach was not suitable for measuring discriminant validity. Henseler et al. (2014), suggested a new approach heterotrait–monotrait (HTMT) to assess the discriminant validity of the instruments. The researchers also applied the HTMT approach to ensure discriminant validity. The threshold value of the HTMT approach should not be above 0.90 (Sarstedt et al., 2017). Table 3 explains that HTMT values were less than threshold value on all constructs. Therefore, it was concluded that the scales fulfilled the requirements of discriminant validity.

**TABLE 3:** Discriminant validity.

Constructs	English language learning	Self- concept	Self- efficacy	Self- imagination
English language learning	0.803			
Self-concept	0.548	0.856		
Self-efficacy	0.368	0.555	0.822	
Self-imagination	0.515	0.798	0.588	0.782

The collinearity problems are required to address in structural equation modeling data analysis. The variance inflation factor (VIF) threshold value should not be above 5 (Hair et al., 2019). In this study, the VIF value is less than 5, and the range of VIF values is between 1.577 and 3.005. This indicates that there was no collinearity among the dimensions used in this study. The model fit indices measures based on three main indicators such SRMR, NFI, and RMS_theta. The range of SRMR values is between 0 and 1, and less than 0.08 is considered ideal (Huang, 2021). The NFI threshold value range is between 0 and 1; the larger the value of NFI, the better performance is achieved. The value of above 0.9 on NFI is considered a model that fits well (Hu and Bentler, 1998). The RMS_theta is the most suitable indicator for assessing the reflective measurement model. A value less than 0.12 indicates that the model fits well (Huang, 2021). Table 4 indicates that the SRMR value of the assessing model fit 0.091. The value of SRMR for measuring the model appropriateness in this study is 0.091. Although this value is higher than the ideal value, it is not much varied and acceptable. The value of NFI is 0.167, which is also acceptable for measuring the appropriateness of the model. The most important indicator for model fit is RMS_theta. The value of RMS_theta is 127, although, it is a little bit above 0.12. However, it is also adequate. Therefore, it was concluded that it was realistically well fit. The collinearity and model fit indicators values are presented in detail in Table 4.

**TABLE 4:** Collinearity and model fit.

Constructs	VIF-ELLO	VIF-SE	Model fit	
Self-concept	2.841	2.754	SRMR	0.091
Self-efficacy (SE)	1.577		NFI	0.820
Self-imagination	3.005	2.754	rms Theta	0.127

*VIF, Variance Inflation Factor; ELLO, English language learning outcomes.*

The explanatory power of the model is measured based on the *R*^2^ value. The range of values for *R*^2^ is from 0 to 1. The higher *R*^2^ value shows high explanatory power, and the lower *R*^2^ value shows lower explanatory power of the model. The threshold values of up to 0.25, 0.50, and 0.75 are considered as weak, moderate, and high explanatory power of the model, respectively. Table 5 shows that English language learning has a *R*^2^ value of 0.319 and a self-efficacy 0.366, and both have moderate explanatory power of the model. The details are presented in Table 5.

**TABLE 5:** R square.

Constructs	R square	R square adjusted
English language learning	0.319	0.318
Self-efficacy	0.366	0.365

### Structure Equation Modeling

#### Hypotheses Testing

We used SmartPLS 3.3.3 bootstrapping mechanism to test the hypotheses. Table 6 indicates the direct relations, coefficients, mean, standard deviations, and t-values along with *P*-values. In Table 6, the outcomes indicate that self-concept has a significant positive relationship with ELLO (β = 0.365, *p* < 0.05), which approves our hypothesis H1. Likewise, self-imagination has a positive and significant relationship with ELLO (β = 0.196, *p* < 0.05), which approves our hypothesis H2. Similarly, self-efficacy has a positive and significant relationship with ELLO (β = 0. 0.059, *p* < 0.05), which approved our hypothesis H3. Two control variables, namely gender and student academic scores, were also measured. Of these, gender indicated a significant impact on ELLO (β = 0. 0.46, *p* < 0.05) (see Table 6).

**TABLE 6:** Direct relations.

Direct relations	Coefficients	Mean	SD	T statistics	P values	Results
Self-Concept - > English Language Learning Outcomes	0.365	0.367	0.035	10.371	0.000	Accepted
Self-Imagination - > English Language Learning Outcomes	0.196	0.195	0.034	5.734	0.000	Accepted
Self-Efficacy - > English Language Learning Outcomes	0.059	0.054	0.022	2.660	0.018	Accepted
**Control Variables**						
Gender	0.046	0.046	0.016	2.852	0.004	
Scores	0.019	0.019	0.017	1.149	0.251	

*SD, standard deviation.*

#### Mediated Effects

**FIGURE 2:**
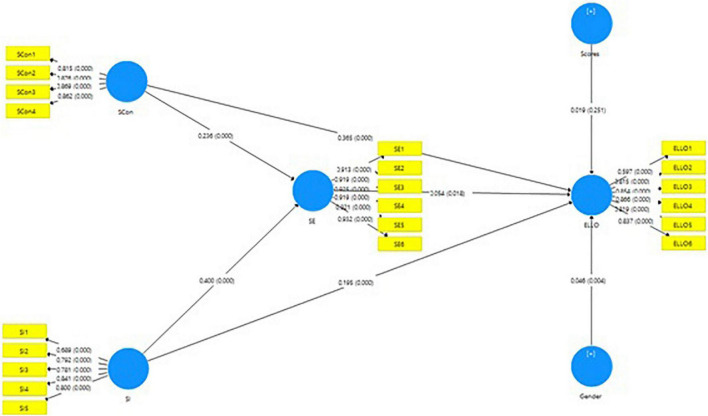
Research out put model.

We measured the mediating role of self-efficacy in the relationship between self-concept and ELLO (β = 0.014, *p* < 0.05), which approves hypothesis H4. Furthermore, self-imagination has a positive and significant relationship with ELLO. Similarly, self-imagination also has a significant and positive relationship with ELLO (β = 0.024, *p* < 0.05), which approves our hypothesis H5. Moreover, Figure 2 and Table 7 present detailed information regarding indirect relations used in the model.

**TABLE 7:** Indirect relations.

Indirect relations	Coefficients	Mean	SD	T statistics	P values	Results
Self-concept - > Self-efficacy - > English language learning outcomes	0.014	0.014	0.006	2.501	0.012	Accepted
Self-imagination - > Self-efficacy - > English language learning outcomes	0.024	0.023	0.009	2.562	0.010	Accepted

*SD, standard deviation.*

## Discussion

The current study adds to the literature of educational psychology by exploring the relationship between self-concept, self-imagination, self-efficacy, and ELLO among students enrolled in blended learning courses in the universities of an emerging country, China, during the COVID-19 pandemic. The results expressed the valuable relationships between self-concept, self-imagination, and self-efficacy on ELLO based on synthesized research framework. As per the knowledge of the authors, most studies of this nature have been conducted in advanced countries, and before the COVID-19 pandemic, very limited studies were done in emerging countries like China to explore the direct and indirect influence of self-concept and self-imagination on ELLO, particularly self-efficacy used as a mediator.

First, the present concentrated on measuring the direct connection of self-concept with ELLO, and the results show that self-concept has a positive and significant connection with ELLO, which approves our hypothesis H1. Prior studies indicated that self-concept has a positive and significant relationship with ELLO (Huang, 2021). Awan et al. (2011) and Du (2012) explored the association between self-concept and learning languages in the Chinese context and the results indicated that English language self-concept predicted students’ ELLO. The possible reason for positive relationship between self-concept in ELLO could be that students have task-specific or domain-specific behaviors and attributes toward ELLO. This relationship indicated that self-concept is a predictor of ELLO. The results can also explain as self-concept has a positive role to increase the ELLO. It can be described that despite the hardships in increasing interfaces in online classes, students worked hard to overcome the challenges by using self-concept and confining in mind their needs to enhance ELLO during the COVID-19 pandemic.

Second, this study explored the direct relationship between self-imagination and ELLO, and the results indicated that self-imagination has a positive and significant influence on ELLO, which supports our hypothesis H2. Prior studies also supported our results self-imagination associated with ELLO (Chao et al., 2019). Similarly, Al-Shehri (2009a) explained that self-imagination is a predictor of learning. The plausible reason for this positive relationship in students might be that the self-imagination encourages students’ cognitive process so that they can understand situation and improve their ELLO. Similarly, self-appraisal can provide foundations in online distance learning situations through meta-cognition in improving learning among learners (Rivers et al., 2021). Self-imagination in English courses proved to be a significant predictor of ELLO.

Third, we investigated the direct influence of self-efficacy on ELLO; the results show that self-efficacy is positively and significantly related to English learning outcomes, which supports our indentations in hypothesis H3. Prior studies support our results (Susanto and Bahar, 2020). Sardegna et al. (2018) conducted a study to explore the connections between self-efficacy and language learning context. The self-efficacy association with English language learning proved to be positive and significant. Self-efficacy is considered the most effective predictor in the context of ELLO. Self-efficacy worked as an effective predictor of ELLO, among blended learning students during the COVID-19 pandemic.

Fourth, in current study, we explored the intervening role of self-efficacy in the relationship between self-concept and ELLO. The results showed that self-efficacy has a mediating role in the relationship between self-concept and ELLO, which approved our hypothesis H4. Prior studies also support our results that language self-efficacy and self-concept enhance ELLO (Noorollahi, 2021). Jaekel (2020) investigated the role of language self-efficacy and self-concept roles to enhance ELLO. Results showed that language self-efficacy worked more effectively with self-concept to play a positive role in enhancing ELLO. Another study indicated that self-efficacy along with self-interest has a strong influence on ELLO in the Asian context (Bai et al., 2020). It can be said that self-efficacy along with self-concept is a predictor of ELLO.

Fifth, the present study investigated the role of self-efficacy between self-imagination and ELLO. The results show that self-efficacy played a mediating role between self-imagination and English learning outcomes and that confirmed our hypothesis H4. Previous studies also supported our results that self-imagination indirectly improved ELLO (Murray, 2011). Al-Shehri (2009a) measured the association between the Ideal Language 2 Self-Imagination. The results indicated that self-imagination and self-efficacy are the predictors of ELLO. Thus, it was proved that self-efficacy had an intervening role in strengthening the relationship between self-concept, self-imagination, and ELLO.

## Conclusion

In this study, we developed a synthesized research model by illustrating the previous literature’s understandings regarding theoretical approaches. The results approved the association between self-concept, self-imagination, self-efficacy, and ELLO. Therefore, it was concluded that self-concept has a direct, positive, and significant role to improve ELLO. Similarly, it was also concluded that self-imagination has a direct, positive, and significant effect on ELLO. Correspondingly, results indicate that self-efficacy has a direct, positive, and significant influence on ELLO. Furthermore, our results indicate that self-concept has an indirect, positive, and significant relationship with ELLO through self-efficacy. Moreover, our results concluded that self-imagination has an indirect, positive, and significant influence on ELLO through self-efficacy.

Our results could be interpreted as follows; self-concept may be considered a determinant of ELLO among students enrolled in blended learning courses during the COVID-19 pandemic. Similarly, self-imagination proved a predictor of ELLO. Furthermore, self-efficacy was demonstrated as a positive factor which could enhance ELLO during this pandemic. Moreover, self-efficacy has an intervening role in strengthening the relationship between self-concept, self-imagination, and ELLO. The interrelationships of self-concept, self-imagination, and self-efficacy in the learning domain provide evidence for shaping the English language learning.

### Practical Implications

The present research results provide signpost of some meaningful, practical, and psychological implications that would enhance English language learning. Teachers should be very careful about the student level of self-concept, self-imagination, and self-efficacy, which are the major predictors of enhancing the English language learning attitudes in blended learning courses. It is very important to develop the teachers’ understanding toward students’ self-concept, self-imagination, and self-efficacy to enhance the ELLO. It is recommended that teacher training should be conducted for this purpose. Teachers should be more equipped with basic skills to understand students’ self-concept, self-imagination, and self-efficacy, and professional assistance may be needed to tackle students’ psychological problems during difficult situation, which can be helpful to improve ELLO among students. University management can hire psychological experts such as teacher–counselors who can provide help to teachers to identify the student level of self-concept, self-imagination, and self-efficacy. In this way, the teachers can enhance ELLO in better way in blended learning courses. For students, they may benefit from well-organized courses on how to negotiate with self-concept, self-imagination, and self-efficacy. Curriculum leadership in universities should integrate the curriculum for self-concept, self-imagination, and self-efficacy improvement among undergraduate students. Furthermore, these steps could also improve English language learning among the students enrolled in blended learning courses.

### Limitations With Future Research Directions

Our study has some limitations that affect the results’ interpretations such as the sample of the study came from a single emerging country China. This factor might cause cultural biasness and have a limited scope of the generalizability of the results. More empirical evidence is needed to validate the results from different sites. We collected the data from only undergraduate students; graduates and post-graduate students were not included in the sample; therefore, stakeholders must be careful when they generalized the results. Future researchers can include graduates and postgraduate students as participants in their studies. Besides, the formation processes of self-concept, self-imagination, and self-efficacy in the English language domain may be investigated in blended learning contexts to profile a more detailed understanding of these psychological factors. Based on the knowledge of the formation process, a more rationalized curriculum design for self-perception development may be produced. Therefore, it would also be very interesting to explore the relationship of emotional intelligence as coping strategies with self-concept, self-imagination, self-efficacy, and ELLO among the students enrolled in blended learning courses. Lastly, in this study, we used self-reported technique to collect the data and analyzed it statistically. Future study may use mixed-method technique that can be employed to increase the validity of results.

## Data Availability Statement

The original contributions presented in the study are included in the article/supplementary material, further inquiries can be directed to the corresponding authors.

## Ethics Statement

This study was reviewed and approved by the Ethics Committee of Guangzhou University. The patients/participants provided their written informed consent to participate in this study.

## Author Contributions

All authors listed have made a substantial, direct, and intellectual contribution to the work equally, and approved it for publication.

## Conflict of Interest

The authors declare that the research was conducted in the absence of any commercial or financial relationships that could be construed as a potential conflict of interest.

## Publisher’s Note

All claims expressed in this article are solely those of the authors and do not necessarily represent those of their affiliated organizations, or those of the publisher, the editors and the reviewers. Any product that may be evaluated in this article, or claim that may be made by its manufacturer, is not guaranteed or endorsed by the publisher.

## References

[B1] Adedoyin O. B. Soykan E. (2020). Covid-19 pandemic and online learning: the challenges and opportunities. *Interact. Learn. Environ.* 1–13. 10.1080/10494820.2020.1813180

[B2] Alrefaie Z. Hassanien M. Al-Hayani A. (2020). Monitoring online learning during COVID-19 pandemic; Suggested online learning portfolio (COVID-19 OLP). *MedEdPublish* 9:110. 10.15694/mep.2020.000110.1 33692645

[B3] Al-Shehri A. S. (2009a). “8. Motivation and Vision: the Relation Between the Ideal L2 Self, Imagination and Visual Style,” in *Motivation, language identity and the L2 self*, eds Dörnyei Z. Ushioda E. (Bristol, UK: Multilingual Matters), 164–171. 10.21832/9781847691293-009

[B4] Al-Shehri A. S. (2009b). “Motivation and Vision: the Relation Between the Ideal L2 Self, Imagination and Visual Style,” in *Motivation, language identity and the L2 self*, eds Dörnyei Z. Ushioda E. (Bristol, UK: Multilingual Matters), 164–171.

[B5] Anyadubalu C. C. (2010). Self-efficacy, anxiety, and performance in the English language among middle-school students in English language program in Satri Si Suriyothai School, Bangkok. *Int. J. Hum. Soc. Sci.* 5 193–198.

[B6] Arens A. K. Jansen M. (2016). Self-concepts in reading, writing, listening, and speaking: a multidimensional and hierarchical structure and its generalizability across native and foreign languages. *J. Educ. Psychol.* 108:646. 10.1037/edu0000081

[B7] Awan R.-U.-N. Noureen G. Naz A. (2011). A Study of Relationship between Achievement Motivation, Self Concept and Achievement in English and Mathematics at Secondary Level. *Int. Educ. Stud.* 4 72–79. 10.3389/fpsyg.2020.533593 33519570 PMC7841336

[B8] Bai B. Nie Y. Lee A. N. (2020). Academic self-efficacy, task importance and interest: relations with English language learning in an Asian context. *J. Multiling. Multicult. Dev.* 1–14. 10.1080/01434632.2020.1746317

[B9] Bai B. Wang J. (2020). The role of growth mindset, self-efficacy and intrinsic value in self-regulated learning and English language learning achievements. *Lang. Teach. Res.* 1–22. 10.1177/1362168820933190

[B10] Baloran E. T. (2020). Knowledge, attitudes, anxiety, and coping strategies of students during COVID-19 pandemic. *J. Loss Trauma* 25 635–642. 10.1080/15325024.2020.1769300

[B11] Bandura A. (1977). *Social learning theory.* Englewood cliffs: Prentice Hall.

[B12] Bandura A. (1997). *Self-Efficacy: the Exercise of Control.* Basingstoke: Macmillan.

[B13] Bandura A. (2006). Guide for constructing self-efficacy scales. *Self Efficacy Beliefs Adolesc.* 5 307–337.

[B14] Bhattacherjee A. Perols J. Sanford C. (2008). Information technology continuance: a theoretic extension and empirical test. *J. Comput. Inf. Syst.* 49 17–26. 10.1080/08874417.2008.11645302

[B15] Biyikl C. (2021). The Relationship between Language Learning Strategies and Academic Self-Concept. *Int. J. Progress. Educ.* 17 101–123.

[B16] Bodnar S. Cucchiarini C. Strik H. van Hout R. (2016). Evaluating the motivational impact of CALL systems: current practices and future directions. *Comput. Assist. Lang. Learn.* 29 186–212.

[B17] Bong M. Skaalvik E. M. (2003). Academic self-concept and self-efficacy: how different are they really? *Educ. Psychol. Rev.* 15 1–40.

[B18] Chang M.-M. (2005). Applying self-regulated learning strategies in a web-based instruction—an investigation of motivation perception. *Comput. Assist. Lang. Learn.* 18 217–230. 10.1080/09588220500178939

[B19] Chao C. N. G. (2013). *Motivational beliefs in language learning of secondary school students in Hong Kong: the relationships among socio-cultural influences, self-efficacy, self-concept, fear of failure and academic achievement.* Ph.D. thesis. Hong Kong: The Education University of Hong Kong.

[B20] Chao C. N. G. McInerney D. M. Bai B. (2019). Self-efficacy and self-concept as predictors of language learning achievements in an Asian bilingual context. *Asia Pac. Educ. Res.* 28 139–147. 10.1007/s40299-018-0420-3

[B21] Chen B. H. Chiu W.-C. Wang C.-C. (2015). The relationship among academic self-concept, learning strategies, and academic achievement: a case study of national vocational college students in Taiwan *via* SEM. *Asia Pac. Educ. Res.* 24 419–431. 10.1007/s40299-014-0194-1

[B22] Chen J. Zhang L. J. (2019). Assessing student-writers’ self-efficacy beliefs about text revision in EFL writing. *Assess. Writ.* 40 27–41. 10.1016/j.asw.2019.03.002

[B23] Chen M. Chai C.-S. Jong M. S.-Y. Chao G. C.-N. (2021). Modeling learners’ self-concept in Chinese descriptive writing based on the affordances of a virtual reality-supported environment. *Educ. Inf. Technol.* 26 6013–6032. 10.1007/s10639-021-10582-4

[B24] Conway M. A. Loveday C. (2015). Remembering, imagining, false memories & personal meanings. *Conscious. Cogn.* 33 574–581. 10.1016/j.concog.2014.12.002 25592676

[B25] Dörnyei Z. (2005). *The Psychology of the Language Learner: individual Differences in Second Language Acquisition.* Mahwah, NJ, US: Lawrence Erlbaum Associates Publishers.

[B26] Dörnyei Z. (2009). “The L2 motivational self system,” in *Motivation, language identity and the L2 self*, eds Dörnyei Z. Ushioda E. (Bristol: Multilingual Matters).

[B27] Dörnyei Z. Ushioda E. (2009). *Motivation, language identity and the L2 self.* Bristol, UK: Multilingual Matters.

[B28] Du M. (2012). A Study of the Relationship between English Self-concept and Language Learning Strategies. *J. Lang. Teach. Res.* 3 508–517.

[B29] Erten I. H. Burden R. L. (2014). The relationship between academic self-concept, attributions, and L2 achievement. *System* 42 391–401. 10.1016/j.system.2014.01.006

[B30] Fornell C. Larcker D. F. (1981). *Structural equation models with unobservable variables and measurement error: algebra and statistics.* Los Angeles, CA: Sage Publications Sage CA.

[B31] Fryer L. K. (2015). Predicting self-concept, interest and achievement for first-year students: the seeds of lifelong learning. *Learn. Individ. Differ.* 38 107–114. 10.1016/j.lindif.2015.01.007

[B32] Gecas V. (1982). The self-concept. *Annu. Rev. Sociol.* 8 1–33. 10.2147/PRBM.S77402 27069373 PMC4818058

[B33] Genc G. Kulusakli E. Aydin S. (2016). Exploring EFL learners’ perceived self-efficacy and beliefs on English language learning. *Aust. J. Teach. Educ.* 41 53–68. 10.3389/fpsyg.2021.801315 35069394 PMC8781971

[B34] Grilli M. D. Glisky E. L. (2011). The self-imagination effect: benefits of a self-referential encoding strategy on cued recall in memory-impaired individuals with neurological damage. *J. Int. Neuropsychol. Soc.* 17 929–933. 10.1017/s1355617711000737 21729405 PMC8620128

[B35] Grilli M. D. McFarland C. P. (2011). Imagine that: self-imagination improves prospective memory in memory-impaired individuals with neurological damage. *Neuropsychol. Rehabil.* 21 847–859. 10.1080/09602011.2011.627263 22150451 PMC3296226

[B36] Guo J.-P. Yang L.-Y. Zhang J. Gan Y.-J. (2021). Academic self-concept, perceptions of the learning environment, engagement, and learning outcomes of university students: relationships and causal ordering. *High. Educ.* 12 1–20. 10.1007/s10734-021-00705-8

[B37] Hair J. F. Risher J. J. Sarstedt M. Ringle C. M. (2019). When to use and how to report the results of PLS-SEM. *Eur. Bus. Rev.* 31 2–24. 10.1108/EBR-11-2018-0203

[B38] Henderson M. Huang H. Grant S. Henderson L. (2012). The impact of Chinese language lessons in a virtual world on university students’ self-efficacy beliefs. *Australas. J. Educ. Technol.* 28 400–419.

[B39] Henseler J. Dijkstra T. K. Sarstedt M. Ringle C. M. Diamantopoulos A. Straub D. W. (2014). Common beliefs and reality about PLS: comments on Rönkkö and Evermann (2013). *Organ. Res. Methods* 17 182–209.

[B40] Hu L.-T. Bentler P. M. (1998). Fit indices in covariance structure modeling: sensitivity to underparameterized model misspecification. *Psychol. Methods* 3:424.

[B41] Huang C.-H. (2021). Using PLS-SEM Model to Explore the Influencing Factors of Learning Satisfaction in Blended Learning. *Educ. Sci.* 11:249. 10.3390/educsci11050249

[B42] Ignatova O. Kalyuga S. Sweller J. (2020). The imagination effect when using textual or diagrammatic material to learn a second language. *Lang. Teach. Res.* 1–21. 10.1177/1362168820971785

[B43] Iqbal J. Qureshi N. Ashraf M. A. Rasool S. F. Asghar M. Z. (2021). The Effect of Emotional Intelligence and Academic Social Networking Sites on Academic Performance During the COVID-19 Pandemic. *Psychol. Res. Behav. Manage.* 14 905–920. 10.2147/PRBM.S316664 34234587 PMC8254613

[B44] Jaekel N. (2020). Language learning strategy use in context: the effects of self-efficacy and CLIL on language proficiency. *Int. Rev. Appl. Linguist. Lang. Teach.* 58 195–220. 10.1515/iral-2016-0102

[B45] Khan R. Jahan A. Sultana S. NaushaadKabir M. M. Haider M. (2021). Accessing Online Instruction amidst COVID-19 in Bangladesh: barriers and Coping Strategies. *Lang. Teach. Res. Q.* 22 33–48.

[B46] Kim D.-H. Wang C. Ahn H. S. Bong M. (2015). English language learners’ self-efficacy profiles and relationship with self-regulated learning strategies. *Learn. Individ. Differ.* 38 136–142.

[B47] Kirmizi Ö (2015). The interplay among academic self-concept, self-efficacy, self-regulation and academic achievement of higher education L2 learners. *Yükseköğretim ve Bilim Dergisi* 5 32–40. 10.5961/jhes.2015.107

[B48] Lai C. Gu M. (2011). Self-regulated out-of-class language learning with technology. *Comput. Assist. Lang. Learn.* 24 317–335.

[B49] Lauermann F. ten Hagen I. (2021). Do teachers’ perceived teaching competence and self-efficacy affect students’ academic outcomes? A closer look at student-reported classroom processes and outcomes. *Educ. Psychol.* 56 265–282.

[B50] Lian J. Chai C. S. Zheng C. Liang J.-C. (2021). Modelling the Relationship Between Chinese University Students’ Authentic Language Learning and Their English Self-efficacy During the COVID-19 Pandemic. *Asia Pac. Educ. Res.* 30 217–228.

[B51] Markus H. Wurf E. (1987). The dynamic self-concept: a social psychological perspective. *Annu. Rev. Psychol.* 38 299–337. 10.1146/annurev.ps.38.020187.001503

[B52] Marsh H. W. (2006). *Self-concept theory, measurement and research into practice: the role of self-concept in educational psychology.* London: British Psychological Society.

[B53] Marsh H. W. Hattie J. (1996). “Theoretical perspectives on the structure of self-concept,” in *Handbook of self-concept: developmental, social, and clinical considerations*, ed. Bracken B. A. (Hoboken: John Wiley & Sons), 38–90.

[B54] Marsh H. W. Martin A. J. Hau K.-T. (2006). “A Multimethod Perspective on Self-Concept Research in Educational Psychology: a Construct Validity Approach,” in *Handbook of multimethod measurement in psychology*, eds Eid M. Diener E. (Washington, DC: American Psychological Association), 441–456.

[B55] Mercer S. (2011). *). Towards an understanding of language learner self-concept.* Berlin/Heidelberg, Germany: Springer Science & Business Media.

[B56] Moore C. Oosthuizen M. P. (1997). “The self concept theory of Carl Rogers,” in *Personality Theories*, eds Rapmund V. Moore C. Oosthuizen P. Shantall T. Dyk A. V. Viljoen H. (Pretoria: University of South Africa), 149–182.

[B57] Moyer A. (2018). An Advantage for Age? Self-Concept and Self-Regulation as Teachable Foundations in Second Language Accent. *CATESOL J.* 30 95–112.

[B58] Murray G. (2011). “Metacognition and imagination in self-access language learning,” in *Fostering autonomy in language learning*, ed. Gardner D. (Gaziantep: Zirve University), 5–16.

[B59] Noorollahi N. (2021). On the Relationship between Iranian English Language Teaching Students’ Self-Efficacy, Self-Esteem, and Their Academic Achievement. *Lang. Teach. Res. Q.* 21 84–96.

[B60] Oxford R. (2017). Exploring psychology in language learning and teaching. *Elt J.* 71 522–524.

[B61] Oyserman D. Markus H. R. (1990). Possible selves and delinquency. *J. Pers. Soc. Psychol.* 59:112. 10.1037//0022-3514.59.1.112 2213484

[B62] Ozfidan B. Machtmes K. L. Demir H. (2014). Socio-Cultural Factors in Second Language Learning: a Case Study of Adventurous Adult Language Learners. *Eur. J. Educ. Res.* 3 185–191. 10.12973/eu-jer.3.4.185

[B63] Pajares F. (2002). Gender and perceived self-efficacy in self-regulated learning. *Theory Pract.* 41 116–125. 10.1207/s15430421tip4102_8 33486653

[B64] Pajares F. Schunk D. H. (2001). Self-beliefs and school success: self-efficacy, self-concept, and school achievement. *Perception* 11 239–266.

[B65] Rivers D. J. Vallance M. Nakamura M. (2021). Metacognitive Knowledge and the Self as Socially Distanced Online Learner: a Virtual Reality Assisted Analysis of Academic Self-Concept. *J. Educ. Technol. Syst.* 50 87–111. 10.1177/0047239521999779

[B66] Rogers C. M. Smith M. D. Coleman J. M. (1978). Social comparison in the classroom: the relationship between academic achievement and self-concept. *J. Educ. Psychol.* 70:50. 632402

[B67] Sabirova E. G. Zaripova Z. F. Mikhaylovsky M. N. Serebrennikova Y. V. Torkunova J. V. Buslaev S. I. (2020). Recreating Imagination and Self-Regulation as Means of Mathematical Thinking Development in Inclusive Education. *EURASIA J. Math. Sci. Technol. Educ.* 16:em1890. 10.29333/ejmste/8501

[B68] Sardegna V. G. Lee J. Kusey C. (2018). Self-efficacy, attitudes, and choice of strategies for English pronunciation learning. *Lang. Learn.* 68 83–114. 10.1111/lang.12263

[B69] Sarstedt M. Ringle C. M. Hair J. F. (2017). Partial least squares structural equation modeling. *Handbook Mark. Res.* 26 1–40. 10.1007/978-3-319-71691-6_1

[B70] Schunk D. H. Pajares F. (2002). “The Development of Academic Self-Efficacy,” in *Development of Achievement Motivation*, eds Wigfield A. Eccles J. S. (San Diego: Academic Press), 15–31. 10.1016/b978-012750053-9/50003-6

[B71] Shen D. Cho M.-H. Tsai C.-L. Marra R. (2013). Unpacking online learning experiences: online learning self-efficacy and learning satisfaction. *Internet High. Educ.* 19 10–17. 10.1016/j.iheduc.2013.04.001

[B72] Shin M.-H. (2018). Effects of project-based learning on students’ motivation and self-efficacy. *English Teach.* 73 95–114. 10.1348/000709907X218160 17588293

[B73] Stracke E. (2016). Language learning strategies of Indonesian primary school students: in relation to self-efficacy beliefs. *System* 60 1–10. 10.1016/j.system.2016.05.001

[B74] Su Y. Zheng C. Liang J.-C. Tsai C.-C. (2018). Examining the relationship between English language learners’ online self-regulation and their self-efficacy. *Australas. J. Educ. Technol.* 34 105–121.

[B75] Susanto A. Bahar H. (2020). “The Effect of Self-Concept and Student Learning Motivation on Learning Achievement of Social Science,” in *International Conference on Community Development (ICCD 2020)*, (Dordrecht: Atlantis Press), 44–47.

[B76] Taguchi T. Magid M. Papi M. (2009). “The L2 motivational self system among Japanese, Chinese, and Iranian learners of English: a comparative study,” in *Motivation, Language Identity and the L2 Self*, eds Dörnyei Z. Ushioda E. (Bristol: Multilingual Matters), 66–97. 10.21832/9781847691293-005

[B77] Tsui A. B. Tollefson J. W. (2017). “1 Language Policy and the Construction of National Cultural Identity,” in *Language policy, culture, and identity in Asian contexts*, eds Tsui A. B. Tollefson J. W. (Boca Raton: Routledge), 1–22.

[B78] Waddington J. (2019). Developing primary school students’ foreign language learner self-concept. *System* 82 39–49. 10.1016/j.system.2019.02.012

[B79] Wang C. Kim D.-H. Bai R. Hu J. (2014). Psychometric properties of a self-efficacy scale for English language learners in China. *System* 44 24–33. 10.1016/j.system.2014.01.015

[B80] Wang C.-H. Shannon D. M. Ross M. E. (2013). Students’ characteristics, self-regulated learning, technology self-efficacy, and course outcomes in online learning. *Distance Educ.* 34 302–323. 10.1080/01587919.2013.835779

[B81] Woodrow L. (2011). College English writing affect: self-efficacy and anxiety. *System* 39 510–522.

[B82] Wylie R. C. (1974). *The self-concept: theory and research on selected topics.* Lincoln, Nebraska: U of Nebraska Press.

[B83] Yang X. Zhou X. Hu J. (2021). Students’ preferences for seating arrangements and their engagement in cooperative learning activities in college English blended learning classrooms in higher education. *High. Educ. Res. Dev.* 1–16. 10.1080/07294360.2021.1901667

[B84] Zhao Y. Wu J. Yang H. Yin X. Li D. Qiu L. (2021). Factors associated with childbirth self-efficacy: a multicenter cross-sectional study in China. *Midwifery* 93:102883. 10.1016/j.midw.2020.102883 33246143

[B85] Zimmerman B. J. (1983). “Social learning theory: a contextualist account of cognitive functioning,” in *Recent advances in cognitive-developmental theory*, ed. Brainerd C. J. (New York: Springer), 1–50 10.1007/978-1-4613-9490-7_1

